# Not all heroes wear capes: The role of movement behaviors in mitigating mortality in cancer survivors and cognitive decline in older adults

**DOI:** 10.1016/j.jnha.2025.100646

**Published:** 2025-08-04

**Authors:** Bruno Remigio Cavalcante, Mariana Ferreira de Souza, Rodrigo Cappato de Araújo

**Affiliations:** a Department of Physical Education, Clinical Exercise Laboratory (LABEC), Universidade Federal do Vale do São Francisco (UNIVASF), Petrolina, PE, Brazil; b Graduate Program in Physical Education (PPGEF), Universidade Federal do Vale do São Francisco (UNIVASF), Petrolina, PE, Brazil; c Graduate Program in Rehabilitation and Funcional Performance (PPGRDF), Universidade de Pernambuco (UPE), Petrolina, PE, Brazil; d Department of Physical Therapy, Universidade de Pernambuco (UPE), Petrolina, PE, Brazil

Cancer is a diverse group of diseases defined by the uncontrolled proliferation of cells that have acquired the ability to evade normal regulatory mechanisms governing cell growth, division, and death [1]. Cancer mortality has declined steadily over recent decades, a trend attributed to advances in early detection, reductions in tobacco use, and improvements in cancer therapies, including the adoption of optimized treatment protocols [2]. As a result, the number of cancer survivors—currently estimated at 18.6 million—is projected to surpass 22 million people by 2035 [3]. These individuals often face complex long-term challenges, resulting from the disease itself, pharmacological treatments, comorbid conditions, and age-related vulnerabilities [4]. The synergistic adverse effects of age, cancer treatments, and related sequelae further increase the overall burden of cancer [4].

**Fig. 1:**
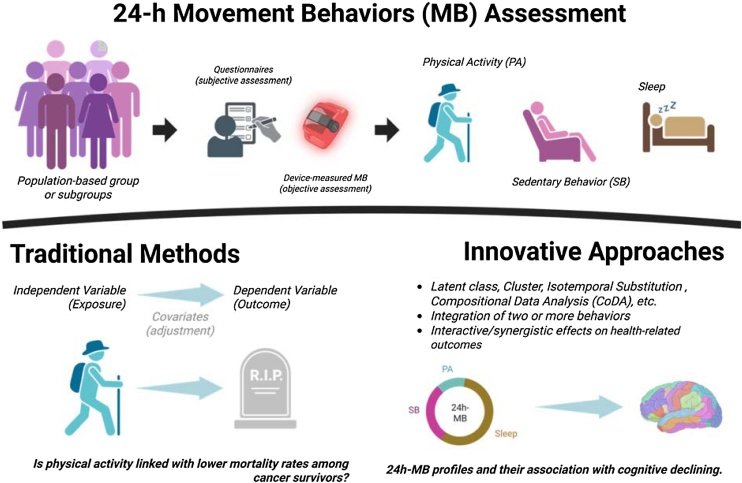
Assessing 24-h movement behaviors: analytical approaches and links to health outcomes (created with Biorender).

Simultaneously, cognitive impairment (e.g., mild cognitive impairment and dementia) continues to impose a growing burden on aging societies. According to the recent World Alzheimer’s Disease Report [5], over 55 million people worldwide were living with dementia in 2020, with a new case diagnosed every three seconds. This number is projected to nearly double every 20 years, reaching 139 million by 2050. No effective drug capable of slowing the rate of cognitive decline in individuals at risk of clinically significant deterioration is currently available [6]. The growing evidence highlights the potential of targeting modifiable risk factors across the life course as a promising strategy for preventing this devastating condition [6].

In this context, movement behaviors (MB), encompassing physical activity (PA), sedentary behavior (SB), and sleep, have gained recognition as critical determinants in the prevention and management of chronic conditions, such as cancer and cognitive decline, and are increasingly acknowledged as integral components of health across the life course [[7], [8], [9], [10], [11]]. Traditionally, each of these behaviors is assessed in isolation (e.g., PA, SB, or sleep as independent variables and health parameters as outcomes in the statistical modeling) with partial adjustment for time in other behaviors [7]. While this approach has merit, it does not fully reflect the reality of how individuals live their daily routine, as time spent in one behavior necessarily affects the others (e.g., poor sleep likely impacts physical activity, and vice versa) [7,12,13]. Therefore, evidence from traditional and innovative approaches might shed important insights regarding the role of 24h-movement behaviors on outcomes of interest [14].

In the present issue of the *Journal of Nutrition, Health and Aging* (JNHA), two prospective observational studies [15,16] offer timely insights into how daily movement behaviors relate to critical health outcomes—namely, mortality in cancer survivors and cognitive decline in community-dwelling adults.

Jiang and colleagues [16] examined the association between accelerometer-measured physical activity and mortality risk among over 11,000 cancer survivors from the UK Biobank. Over a median follow-up of 8.9 years, higher levels of moderate to vigorous physical activity (MVPA) were significantly associated with reduced all-cause and cancer-specific mortality. The authors observed a dose-response relationship, with risk reductions of up to 48% for individuals accumulating greater minutes of MVPA per week. Cancer-specific mortality of cancers originating from breast, male genital system, digestive system and blood system in participants with higher time spent in MVPA were also reduced by 43%–59%. Notably, only marginal significant survival benefit was found for light-intensity physical activity.

In a second study, Palazuelos-González and colleagues [15] aimed to examine how distinct movement behavior patterns combining sleep, physical activity, and sedentary behavior were associated with cognitive performance over time. To that end, they applied latent class analysis to identify behavioral profiles based on self-reported sleep, physical activity, and sedentary time among more than 12,000 middle-aged and older adults (aged 45–86 years) from the Canadian Longitudinal Study on Aging (CLSA). Cognitive function was assessed through a comprehensive neuropsychological battery over a three-year follow-up. 24h-MB were ascertained subjectively throughout validated questionnaires. The main findings revealed three behavioral classes: Sedentary/Disturbed Sleep (∼54%), Intermediately Active/Normal Sleep (∼35%), and Active/Normal Sleep (∼11%). Multivariate regression models demonstrated that Sedentary/Disturbed sleepers had greater cognitive decline after a 3-year follow-up compared with the intermediate profile. Interestingly, the Active/Normal sleepers also experienced a greater decline in memory and overall cognition compared to the intermediate group, suggesting a non-linear association between activity intensity and cognitive outcomes. The authors concluded that, in mid- and later life, sleep disturbances tend to co-occur with sedentary behavior, and moderate physical activity appears most effective in supporting healthy cognitive aging, compared to either sedentary behavior or excessive physical activity.

Altogether, the findings underscore the importance of movement behaviors in shaping health trajectories in later life and cancer survivorship, with significant public health implications. Rather than focusing solely on vigorous or prolonged activity, their findings suggest that balanced daily movement patterns, especially those integrating moderate activity and regular sleep, may help mitigate mortality risk and cognitive deterioration. Despite these advances, important knowledge gaps remain. Further research is needed to move the field forward. (1) Innovative analytical methods, including isotemporal substitution modeling and compositional data analysis (CoDA), are now available and increasingly used. These approaches may foster significant advances in understanding how 24-h movement behavior interact and exert synergistic effects on health outcomes [7]; (2) More longitudinal data using device-measured 24-h movement behavior are imperative to better understand their impact on health-related outcomes throughout the aging process; (3) Well-designed and adequately powered randomized clinical trials are also warranted to confirm the preventive and therapeutic efficacy of both independent and combined behavioral interventions, as well as to provide reliable evidence to support 24h-MB guidelines and clinical decision-making.

In summary, although neither Palazuelos-González [10] nor Jiang [11] evaluated formal interventions, both studies offer compelling evidence that daily behaviors—often overlooked in clinical contexts—play a crucial role in determining long-term health outcomes. In the journey toward healthier aging and improved cancer survivorship, movement behaviors may not wear capes, but they certainly act as silent guardians.

## Declaration of competing interest

We have no conflicts of interest to disclose.
